# Supplementary material for: HIVprotI: an integrated web based platform for prediction and design of HIV proteins inhibitors
Source: J Cheminform. 2018 Mar 9;10:12. doi: 10.1186/s13321-018-0266-y (PMC5845081; doi:10.1186/s13321-018-0266-y)
Supplement: Supplementary file 2 — Additional file 2. Source code of HIVProtI web server. [file 13321_2018_266_MOESM2_ESM.zip › HIVprotI_Source-code/hivprotI/weka-3-6-6/doc/weka/gui/CheckBoxList.html]

CheckBoxList


---


|  |  |  |
| --- | --- | --- |
| **Overview** \| **Package** \| **Class** \| **Tree** \| **Deprecated** \| **Index** \| **Help** \| **Weka's home** | |  |
| **PREV CLASS**   **NEXT CLASS** | **FRAMES**    **NO FRAMES**     **All Classes** |  |
| SUMMARY: NESTED \| FIELD \| CONSTR \| METHOD | DETAIL: FIELD \| CONSTR \| METHOD |  |


---


## weka.gui Class CheckBoxList

```
java.lang.Object
  java.awt.Component
      java.awt.Container
          javax.swing.JComponent
              javax.swing.JList
                  weka.gui.CheckBoxList
```

**All Implemented Interfaces:**: java.awt.image.ImageObserver, java.awt.MenuContainer, java.io.Serializable, javax.accessibility.Accessible, javax.swing.Scrollable

---

``` public class CheckBoxList extends javax.swing.JList ```

An extended JList that contains CheckBoxes. If necessary a CheckBoxListItem
wrapper is added around the displayed object in any of the Model methods,
e.g., addElement. For methods returning objects the opposite takes place,
the wrapper is removed and only the payload object is returned.

**Version:**
:   $Revision: 7059 $

**Author:**
:   fracpete (fracpete at waikato dot ac dot nz)

**See Also:**: Serialized Form

---

| **Nested Class Summary** | |
| --- | --- |
| `class` | `CheckBoxList.CheckBoxListModel`             A specialized model. |
| `class` | `CheckBoxList.CheckBoxListRenderer`             A specialized CellRenderer for the CheckBoxList |

**Nested classes/interfaces inherited from class javax.swing.JList**

`javax.swing.JList.DropLocation`

**Nested classes/interfaces inherited from class javax.swing.JComponent**

`javax.swing.JComponent.AccessibleJComponent`

**Nested classes/interfaces inherited from class java.awt.Component**

`java.awt.Component.BaselineResizeBehavior`


**Field Summary**

**Fields inherited from class javax.swing.JList**

`HORIZONTAL_WRAP, VERTICAL, VERTICAL_WRAP`

**Fields inherited from class javax.swing.JComponent**

`TOOL_TIP_TEXT_KEY, UNDEFINED_CONDITION, WHEN_ANCESTOR_OF_FOCUSED_COMPONENT, WHEN_FOCUSED, WHEN_IN_FOCUSED_WINDOW`

**Fields inherited from class java.awt.Component**

`BOTTOM_ALIGNMENT, CENTER_ALIGNMENT, LEFT_ALIGNMENT, RIGHT_ALIGNMENT, TOP_ALIGNMENT`

**Fields inherited from interface java.awt.image.ImageObserver**

`ABORT, ALLBITS, ERROR, FRAMEBITS, HEIGHT, PROPERTIES, SOMEBITS, WIDTH`


**Constructor Summary**

`CheckBoxList()`
  
          initializes the list with an empty CheckBoxListModel

`CheckBoxList(CheckBoxList.CheckBoxListModel model)`
  
          initializes the list with the given CheckBoxListModel


| **Method Summary** | |
| --- | --- |
| `boolean` | `getChecked(int index)`             returns the checked state of the element at the given index |
| `int[]` | `getCheckedIndices()`             returns an array with the indices of all checked items |
| `void` | `setChecked(int index, boolean checked)`             sets the checked state of the element at the given index |
| `void` | `setListData(java.lang.Object[] listData)`             Constructs a CheckBoxListModel from an array of objects and then applies setModel to it. |
| `void` | `setListData(java.util.Vector listData)`             Constructs a CheckBoxListModel from a Vector and then applies setModel to it. |
| `void` | `setModel(javax.swing.ListModel model)`             sets the model - must be an instance of CheckBoxListModel |

**Methods inherited from class javax.swing.JList**

`addListSelectionListener, addSelectionInterval, clearSelection, ensureIndexIsVisible, getAccessibleContext, getAnchorSelectionIndex, getCellBounds, getCellRenderer, getDragEnabled, getDropLocation, getDropMode, getFirstVisibleIndex, getFixedCellHeight, getFixedCellWidth, getLastVisibleIndex, getLayoutOrientation, getLeadSelectionIndex, getListSelectionListeners, getMaxSelectionIndex, getMinSelectionIndex, getModel, getNextMatch, getPreferredScrollableViewportSize, getPrototypeCellValue, getScrollableBlockIncrement, getScrollableTracksViewportHeight, getScrollableTracksViewportWidth, getScrollableUnitIncrement, getSelectedIndex, getSelectedIndices, getSelectedValue, getSelectedValues, getSelectionBackground, getSelectionForeground, getSelectionMode, getSelectionModel, getToolTipText, getUI, getUIClassID, getValueIsAdjusting, getVisibleRowCount, indexToLocation, isSelectedIndex, isSelectionEmpty, locationToIndex, removeListSelectionListener, removeSelectionInterval, setCellRenderer, setDragEnabled, setDropMode, setFixedCellHeight, setFixedCellWidth, setLayoutOrientation, setPrototypeCellValue, setSelectedIndex, setSelectedIndices, setSelectedValue, setSelectionBackground, setSelectionForeground, setSelectionInterval, setSelectionMode, setSelectionModel, setUI, setValueIsAdjusting, setVisibleRowCount, updateUI`

**Methods inherited from class javax.swing.JComponent**

`addAncestorListener, addNotify, addVetoableChangeListener, computeVisibleRect, contains, createToolTip, disable, enable, firePropertyChange, firePropertyChange, firePropertyChange, getActionForKeyStroke, getActionMap, getAlignmentX, getAlignmentY, getAncestorListeners, getAutoscrolls, getBaseline, getBaselineResizeBehavior, getBorder, getBounds, getClientProperty, getComponentPopupMenu, getConditionForKeyStroke, getDebugGraphicsOptions, getDefaultLocale, getFontMetrics, getGraphics, getHeight, getInheritsPopupMenu, getInputMap, getInputMap, getInputVerifier, getInsets, getInsets, getListeners, getLocation, getMaximumSize, getMinimumSize, getNextFocusableComponent, getPopupLocation, getPreferredSize, getRegisteredKeyStrokes, getRootPane, getSize, getToolTipLocation, getToolTipText, getTopLevelAncestor, getTransferHandler, getVerifyInputWhenFocusTarget, getVetoableChangeListeners, getVisibleRect, getWidth, getX, getY, grabFocus, isDoubleBuffered, isLightweightComponent, isManagingFocus, isOpaque, isOptimizedDrawingEnabled, isPaintingForPrint, isPaintingTile, isRequestFocusEnabled, isValidateRoot, paint, paintImmediately, paintImmediately, print, printAll, putClientProperty, registerKeyboardAction, registerKeyboardAction, removeAncestorListener, removeNotify, removeVetoableChangeListener, repaint, repaint, requestDefaultFocus, requestFocus, requestFocus, requestFocusInWindow, resetKeyboardActions, reshape, revalidate, scrollRectToVisible, setActionMap, setAlignmentX, setAlignmentY, setAutoscrolls, setBackground, setBorder, setComponentPopupMenu, setDebugGraphicsOptions, setDefaultLocale, setDoubleBuffered, setEnabled, setFocusTraversalKeys, setFont, setForeground, setInheritsPopupMenu, setInputMap, setInputVerifier, setMaximumSize, setMinimumSize, setNextFocusableComponent, setOpaque, setPreferredSize, setRequestFocusEnabled, setToolTipText, setTransferHandler, setVerifyInputWhenFocusTarget, setVisible, unregisterKeyboardAction, update`

**Methods inherited from class java.awt.Container**

`add, add, add, add, add, addContainerListener, addPropertyChangeListener, addPropertyChangeListener, applyComponentOrientation, areFocusTraversalKeysSet, countComponents, deliverEvent, doLayout, findComponentAt, findComponentAt, getComponent, getComponentAt, getComponentAt, getComponentCount, getComponents, getComponentZOrder, getContainerListeners, getFocusTraversalKeys, getFocusTraversalPolicy, getLayout, getMousePosition, insets, invalidate, isAncestorOf, isFocusCycleRoot, isFocusCycleRoot, isFocusTraversalPolicyProvider, isFocusTraversalPolicySet, layout, list, list, locate, minimumSize, paintComponents, preferredSize, printComponents, remove, remove, removeAll, removeContainerListener, setComponentZOrder, setFocusCycleRoot, setFocusTraversalPolicy, setFocusTraversalPolicyProvider, setLayout, transferFocusBackward, transferFocusDownCycle, validate`

**Methods inherited from class java.awt.Component**

`action, add, addComponentListener, addFocusListener, addHierarchyBoundsListener, addHierarchyListener, addInputMethodListener, addKeyListener, addMouseListener, addMouseMotionListener, addMouseWheelListener, bounds, checkImage, checkImage, contains, createImage, createImage, createVolatileImage, createVolatileImage, dispatchEvent, enable, enableInputMethods, firePropertyChange, firePropertyChange, firePropertyChange, firePropertyChange, firePropertyChange, getBackground, getBounds, getColorModel, getComponentListeners, getComponentOrientation, getCursor, getDropTarget, getFocusCycleRootAncestor, getFocusListeners, getFocusTraversalKeysEnabled, getFont, getForeground, getGraphicsConfiguration, getHierarchyBoundsListeners, getHierarchyListeners, getIgnoreRepaint, getInputContext, getInputMethodListeners, getInputMethodRequests, getKeyListeners, getLocale, getLocation, getLocationOnScreen, getMouseListeners, getMouseMotionListeners, getMousePosition, getMouseWheelListeners, getName, getParent, getPeer, getPropertyChangeListeners, getPropertyChangeListeners, getSize, getToolkit, getTreeLock, gotFocus, handleEvent, hasFocus, hide, imageUpdate, inside, isBackgroundSet, isCursorSet, isDisplayable, isEnabled, isFocusable, isFocusOwner, isFocusTraversable, isFontSet, isForegroundSet, isLightweight, isMaximumSizeSet, isMinimumSizeSet, isPreferredSizeSet, isShowing, isValid, isVisible, keyDown, keyUp, list, list, list, location, lostFocus, mouseDown, mouseDrag, mouseEnter, mouseExit, mouseMove, mouseUp, move, nextFocus, paintAll, postEvent, prepareImage, prepareImage, remove, removeComponentListener, removeFocusListener, removeHierarchyBoundsListener, removeHierarchyListener, removeInputMethodListener, removeKeyListener, removeMouseListener, removeMouseMotionListener, removeMouseWheelListener, removePropertyChangeListener, removePropertyChangeListener, repaint, repaint, repaint, resize, resize, setBounds, setBounds, setComponentOrientation, setCursor, setDropTarget, setFocusable, setFocusTraversalKeysEnabled, setIgnoreRepaint, setLocale, setLocation, setLocation, setName, setSize, setSize, show, show, size, toString, transferFocus, transferFocusUpCycle`

**Methods inherited from class java.lang.Object**

`equals, getClass, hashCode, notify, notifyAll, wait, wait, wait`

**Constructor Detail**

### CheckBoxList

```
public CheckBoxList()
```

:   initializes the list with an empty CheckBoxListModel

---


### CheckBoxList

```
public CheckBoxList(CheckBoxList.CheckBoxListModel model)
```

:   initializes the list with the given CheckBoxListModel

    **Parameters:**: `model` - the model to initialize with


**Method Detail**

### setModel

```
public void setModel(javax.swing.ListModel model)
```

:   sets the model - must be an instance of CheckBoxListModel

    :   **Overrides:**: `setModel` in class `javax.swing.JList`
    :   **Parameters:**: `model` - the model to use **Throws:**: `java.lang.IllegalArgumentException` - if the model is not an instance of CheckBoxListModel **See Also:**: `CheckBoxList.CheckBoxListModel`

---


### setListData

```
public void setListData(java.lang.Object[] listData)
```

:   Constructs a CheckBoxListModel from an array of objects and then applies
    setModel to it.

    :   **Overrides:**: `setListData` in class `javax.swing.JList`
    :   **Parameters:**: `listData` - the data to use

---


### setListData

```
public void setListData(java.util.Vector listData)
```

:   Constructs a CheckBoxListModel from a Vector and then applies setModel
    to it.

    :   **Overrides:**: `setListData` in class `javax.swing.JList`

---


### getChecked

```
public boolean getChecked(int index)
```

:   returns the checked state of the element at the given index

    :   **Parameters:**: `index` - the index of the element to return the checked state for **Returns:**: the checked state of the specifed element

---


### setChecked

```
public void setChecked(int index,
                       boolean checked)
```

:   sets the checked state of the element at the given index

    :   **Parameters:**: `index` - the index of the element to set the checked state for: `checked` - the new checked state

---


### getCheckedIndices

```
public int[] getCheckedIndices()
```

:   returns an array with the indices of all checked items

    :   **Returns:**: the indices of all items that are currently checked


---


|  |  |  |
| --- | --- | --- |
| **Overview** \| **Package** \| **Class** \| **Tree** \| **Deprecated** \| **Index** \| **Help** \| **Weka's home** | |  |
| **PREV CLASS**   **NEXT CLASS** | **FRAMES**    **NO FRAMES**     **All Classes** |  |
| SUMMARY: NESTED \| FIELD \| CONSTR \| METHOD | DETAIL: FIELD \| CONSTR \| METHOD |  |


---
